# PaperClip: rapid multi-part DNA assembly from existing libraries

**DOI:** 10.1093/nar/gku829

**Published:** 2014-09-08

**Authors:** Maryia Trubitsyna, Gracjan Michlewski, Yizhi Cai, Alistair Elfick, Christopher E. French

**Affiliations:** 1 School of Biological Sciences, University of Edinburgh, Edinburgh, EH9 3JR, UK; 2 School of Engineering, University of Edinburgh, Edinburgh, EH9 3JL, UK; 3 School of Biological Sciences, Wellcome Trust Centre for Cell Biology, University of Edinburgh, Edinburgh, EH9 3JR, UK

## Abstract

Assembly of DNA ‘parts’ to create larger constructs is an essential enabling technique for bioengineering and synthetic biology. Here we describe a simple method, PaperClip, which allows flexible assembly of multiple DNA parts from currently existing libraries cloned in any vector. No restriction enzymes, mutagenesis of internal restriction sites, or reamplification to add end homology are required. Order of assembly is directed by double stranded oligonucleotides—‘Clips’. Clips are formed by ligation of pairs of oligonucleotides corresponding to the ends of each part. PaperClip assembly can be performed by polymerase chain reaction or by cell extract-mediated recombination. Once multi-use Clips have been prepared, assembly of at least six DNA parts in any order can be accomplished with high efficiency within several hours.

## INTRODUCTION

Assembly of DNA sequences (parts) together is a critical enabling technology for synthetic biology. DNA assembly methods fall into several classes (1). End-homology based methods, such as isothermal (Gibson) assembly (2), CPEC (3), SLiCE (4) and SLIC (5) allow assembly of multiple parts in a single reaction, but require homology between the ends of neighbouring parts. The homology regions must usually be added by polymerase chain reaction (PCR) amplification prior to assembly, requiring new primers and sequence confirmation of products for each assembly. Methods such as MoClo (6), the original Golden Gate (7) and GoldenBraid (8,9), based on Type IIS restriction enzymes generating user-defined overhangs, are rapid but may require mutagenesis of internal restriction sites. Moreover parts must often be cloned in specific vectors to provide the correct restriction sites and overhangs for each location. Specific plasmids are also required for methods based on serine integrases (10). The USER cloning method is independent of restriction enzymes and special vectors, but requires the USER™ enzyme, and dU-containing primers must be obtained for amplification of parts (11,12). BioBrick™-like methods (BBF RFC10) (13) are completely flexible in terms of part order, but require relatively slow pairwise assembly, and all parts must be free of internal sites for the relevant restriction enzymes. Linker based methods such as GenBrick (BBF RFC98) and MODAL (14) overcome some of these issues through a pre-assembly step in which oligonucleotide linkers are ligated to the ends generated by Type IIS restriction enzymes, allowing rapid flexible assembly of multiple parts, but still require libraries to be cloned in specific donor plasmids and to be free of internal sites for the relevant enzymes. A number of recent publications (15,16) have described methods using bridging oligonucleotides to direct assembly of multiple parts. These methods have many advantages, but depending on particular design might require new oligonucleotides to be synthesized for each assembly; thus they may be poorly suited to applications where many parallel assemblies are to be performed. Here we show a new approach to DNA assembly using double stranded bridging oligonucleotides—Clips. For each DNA part to be joined four short oligonucleotides are obtained; these may be reused in any assembly involving this part. The order of the parts in the assembly is controlled by the order of the oligonucleotides ligated to create the Clips. The assembly reaction can be performed using various methods. Here we demonstrate assembly by PCR and by*Escherichia coli* cell extract expressing recombination proteins.

## MATERIALS AND METHODS

### Design of Clip oligonucleotides

**Figure 1.**
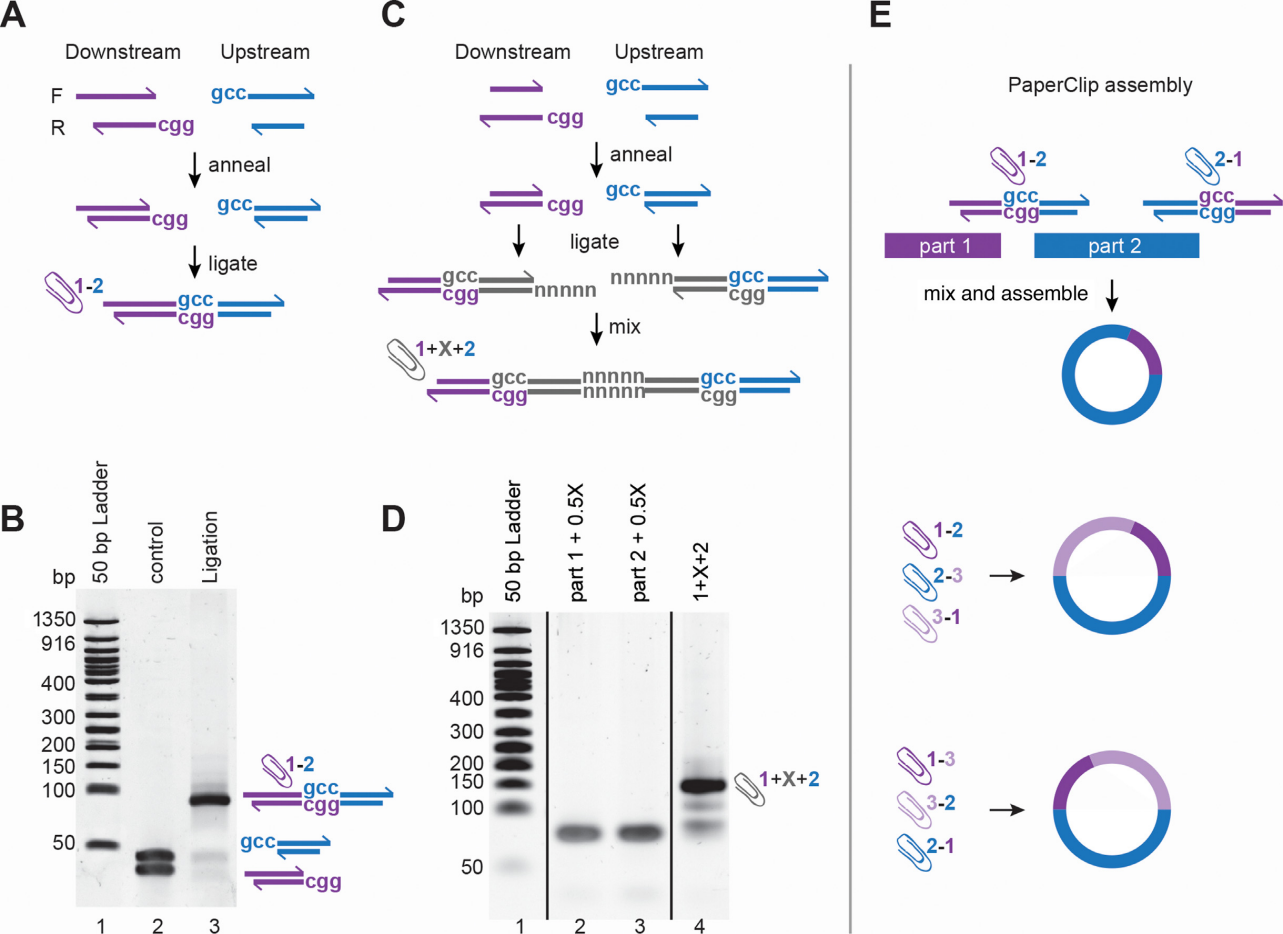
Methods for preparing Clips. (**A**) Scheme of Clip preparation—oligonucleotides corresponding to the downstream region of one part (in purple) and two for the upstream region of the second part (in blue) are annealed pairwise and ligated. Since the inner parts contain GCC sticky ends and the outer ends non-compatible overhangs, the ligation reaction is correct and efficient (**B**). (**C** and **D**) Scheme of addition of an intervening sequence between the Clips. The intervening sequence (in grey) is synthesized in the form of four oligonucleotides, forming GCC and GGC overhangs, which are ligated with the Downstream and Upstream half-Clips respectively. The two reactions are then mixed together and the sequence of interest is restored guided by five base pre-designed overhangs. The efficiency of ligation is sufficient to perform standard PaperClip assembly. (**E**) Schematic diagram of two part assembly using Clips. For two part assembly two DNA parts and two Clips are mixed together and either PCR or cell extract mediated recombination is performed to assemble a circular plasmid. For assemblies with three or more parts, the order of the parts, 1–2–3 or 1–3–2, is guided by the order of the half-Clips. Since all half-Clips have identical GCC overhangs, any order of the parts can be predefined.

For each part to be assembled four oligonucleotides should be obtained: upstream forward (UF) of ∼43 bases; upstream reverse (UR) of ∼37 bases; downstream forward (DF) of ∼40 bases and downstream reverse (DR) of ∼40 bases. The Clip design algorithm contains four simple steps:

- Select the first 40 bases of the forward strand of the part, and add GCC at the 5′-end; this is the UF oligonucleotide.
- Select the first 37 bases, create a reverse complement oligonucleotide; this is the UR oligonucleotide. Annealing of UF and UR forms the Upstream half-Clip with 5′ GCC overhang at the external end, and a three base 3′ overhang at the internal end (Figure 1A).
- Select the last 40 bases of the part, and check that the first three bases are not GCC or GGC. If this condition is met, this is the DF oligonucleotide. If the first three bases are GCC or GGC, then remove the first base and check again.
- Select the last 37 bases (or a sequence three bases shorter than DF). Create a reverse complement oligonucleotide and add GGC at the 5′-end of this sequence. This is the DR oligonucleotide. Annealing of DF and DR forms the Downstream half-Clip with a 5′ overhang of GGC at the external end, and a 5′ overhang which is not GCC or GGC at the internal end (Figure 1A).

### Preparation of half-Clips

Single stranded oligonucleotides were obtained from Sigma, and dissolved in nuclease-free water (Qiagen) to a final concentration of 100 μM. Forward and reverse oligonucleotides were mixed together (final concentration 40 μM each) and phosphorylation was performed in 50 μl final volume with 0.5 μl of T4 polynucleotide kinase (Thermo Scientific) with addition of adenosine triphosphate (ATP) (final concentration 1 mM) for 30 min at 37°C. 5M NaCl was added to final concentration 500 mM and tubes were placed in a thermal cycler for annealing by slow cooling down from 95°C to 4°C at 0.1°C/s (∼20 min). The half-Clips thus formed were stored at −20°C for later use in assemblies as required.

### Preparation of clips

Clips are made by ligation of half-Clips in the desired order. Ligation was performed in 10 μl final volume with addition of ATP (final concentration 1 mM) with 0.5 μl of T4 DNA Ligase (Thermo Scientific). Upstream and Downstream half-Clips (final concentration 14 μM each) were ligated at 16°C for 1 h, followed by heat inactivation of ligase at 65°C for 20 min. Clips were stored at −20°C.

Efficiency of the ligation reaction (0.5 μl ligation mixture + 4.5 μl water + 1 μl 6x Purple Loading Buffer, New England Biolabs) was visualized by polyacrylamide gel electrophoresis on 12% polyacrylamide gels in 0.5x TBE, run at 50 V for 10 min followed by 100 V for 1 h, at room temperature, and stained with with GelGreen™ Nucleic Acid Gel Stain (BIOTIUM Inc.) in water. The DNA bands were visualized with BioRad Gel Doc system.

### Preparation of parts

To cut out a DNA part or to linearize a plasmid vector 1 μg of DNA was subjected to restriction digestion with an appropriate enzyme (New England Biolabs), cutting outside the part sequence, for 1 h. The restriction enzyme was heat inactivated, and the parts were stored at −20°C. For each assembly, 2 μl of the reaction mixture was used.

When required, DNA parts were obtained by amplification from plasmid DNA using UF and DR oligonucleotides of the corresponding part as primers for PCR. PCR reactions were prepared in 50 μl volume using KOD Hot Start DNA polymerase (Novagen) according to the manufacturer's instructions. Generally a standard 2-step reaction was used: 95°C for 2 min, followed by 35 cycles of 95°C for 20 s and 70°C (0.5°C/s) for 20 s/kb. The product was purified by PCR column purification (Qiagen), eluted to a final concentration ∼100 ng/μl, and stored at −20°C. For each assembly 1 μl was used.

### Assembly procedure using PCR

For assembly by PCR, the assembly reaction contained 200 ng of the destination vector backbone pSB1C3 (http://parts.igem.org) (2 kb, 15 nM final concentration), 100 ng of the amplified DNA parts (or 2 μl of the restriction digest reaction), 0.21 μl of the appropriate Clips (60 nM final concentration), 1xKOD Hot Start DNA polymerase buffer (Novagen), 0.2 mM dNTPs, 1.5 mM MgSO_4_ and 1 μl of KOD Hot Start DNA polymerase (Novagen). Two-step PCR was performed: 95°C for 2 min, followed by 20 cycles of 95°C for 20 s and 70°C (0.5°C/s) for 20 s/kb. Increase in the number of cycles was not observed to result in an increase in the number of colonies.

*Escherichia coli* DH10B chemically competent cells (100 μl, 3 × 10^7^ CFU/μg DNA) were transformed with 5 μl (or 1 μl for two part assembly) of the PCR reaction, 400 μl of SOC medium was added and cells were allowed to recover for 80 min at 37°C with 200 rpm agitation, 200 μl of the reaction was plated on LB agar containing kanamycin (50 μg/ml) in duplicates and incubated for 20 h at 37°C. Kanamycin was used for selection, since assemblies contained a kanamycin resistance gene derived from a plasmid with a conditional R6K replication origin, which can not propagate in *E.coli* DH10B (17). From 10 to 2000 kanamycin resistant colonies were typically observed depending on the number of parts assembled.

### Second ‘boost’ PCR to increase the number of colonies obtained

To boost the number of colonies, a second, primer-less PCR reaction was performed: 5 μl of the first reaction, 1xKOD Hot Start DNA polymerase buffer (Novagen), 0.2 mM dNTPs, 1.5 mM MgSO_4_ and 1 μl of KOD Hot Start DNA polymerase (Novagen). No primers are added at this stage of the assembly. Two-step PCR was performed: 95°C for 2 min, 20 cycles: 95°C for 20 s, 70°C (0.5°C/s) for 20 s/kb. This step was used for six-part assembly including green fluorescent protein (GFP) and red fluorescent protein (RFP) as well as the kanamycin-resistance cassette, and was observed to increase the number of kanamycin-resistant colonies obtained from 10 to 1500.

### Assembly procedure using cell extracts

For assembly using cell extracts, the extracts were prepared from *E.coli* DH10B expressing the Red/ET (18) system (GeneBridges). Induction of expression was performed according to the manufacturer's instructions. Overnight cultures of *E.coli* DH10B Red/ET cells were inoculated 1:100 in 250 ml of 2xTY medium containing tetracycline (12 μg/ml) and grown at 30°C until OD600 reached 0.38. The expression of recombination proteins was induced with arabinose (0.3% v/v final concentration) and the culture was incubated for a further 45 min at 37°C with 200 rpm agitation. The cells were pelleted at 5000 *g* for 20 min at 4°C and the pellet (0.37 g) was re-suspended in a final volume of 483 μl buffer D (100 mM Tris pH 8.0, 100 mM KCl, 20% (w/v) glycerol, 0.2 mM ethylenediaminetetraacetic acid (EDTA), 0.5 mM dithiothreitol (DTT), 0.2 mM phenylmethylsulphonyl fluoride (PMSF)) (19). Cell extract was prepared using a Bioruptor single sonicator (Diagenode). The sonication was performed in a 4°C water bath in 1.5 ml microcentrifuge tubes for 5 min using cycles of 30 s ON followed by 30 s OFF. Cell debris was pelleted for 5 min at 10000 *g*, at 4°C, and glycerol was added to a final concentration of 50% (v/v). The final protein concentration of the extract was ∼10 mg/ml. Extracts were stored at −20°C in Eppendorf LoBind 0.5 ml tubes. Alternatively, cell extracts were prepared by chemical lysis according to the original protocol for SLiCE assembly (4).

For the assembly reaction, 130 ng of the destination vector backbone pSB1C3 (2 kb, 10 nM final concentration) was mixed with linear DNA parts in a 1:10 molar ratio and Clips in 1:50 molar ratio, in buffer (4) containing 50 mM Tris pH 7.5, 10 mM MgCl_2_, 1 mM ATP, 1 mM DTT and 2 μl of cell extract, in a final volume of 10 μl. After 1 h incubation at 37°C the whole reaction (10 μl) was used to transform 100 μl of *E.coli* DH10B chemically competent cells. After 80 min recovery at 37°C with 200 rpm agitation, 400 μl (of the 500 μl total volume) was plated on LB agar containing either chloramphenicol (40 μg/ml) (backbone resistance), X-gal (0.1 mM) and isopropyl β-D-1-thiogalactopyranoside (IPTG) (80 μg/ml), or kanamycin (50 μg/ml) and incubated for 20 h at 37°C. From 20 to 200 colonies were obtained depending on the number of parts assembled.

### Analysis of the assembled plasmids

To reduce background due to re-ligated pSB1C3 vector, assemblies included a kanamycin resistance gene from a donor plasmid with an R6K conditional origin of replication (17), which could only be propagated in a host strain expressing pir protein (e.g. *E.coli* S17 lambda-pir). This enabled selection of the clones propagating assembled products using kanamycin as a selection marker, since the plasmid donor of the kanamycin cassette could not replicate in *E.coli* DH10B used for the assembly.

Presence of other selection markers was confirmed by streaking out kanamycin resistant clones on the appropriate antibiotics or LB agar plates containing IPTG (0.1 mM) and X-Gal (80 μg/ml). Plasmid DNA was isolated from 3 to 10 resistant clones (QIAprep Spin Miniprep Kit, Qiagen) and 200 ng of DNA was digested with EcoRV (New England Biolabs) in 10 μl final volume for 1 h. The products of the restriction digest were analyzed by agarose gel electrophoresis on 1% (w/v) agarose gels in 1xTAE, run at 60 V for 60 min at room temperature, using in-gel staining with SaveView (NBS Biologicals). The DNA bands were visualized using BioRad Gel Doc. All interpart regions of the final constructs were sequenced to confirm the presence of the characteristic GCC seams and the absence of mutations. Sequencing data, including sequencing primers and the final constructs can be found at http://archive.bio.ed.ac.uk/papers/paperclip/.

## RESULTS

### Generation of clips

The order of the DNA parts in PaperClip assembly is determined by multi-use bridging double-stranded oligonucleotides, designated ‘Clips’. These Clips are generated in a pre-assembly step by ligation of double-stranded oligonucleotides matching the end of each part (Figure 1A and B). The outer ends of each oligonucleotide pair (half-Clip) bear three base extensions giving the sequence ‘GCC’ to allow ligation of adjacent half-Clips. This will generate a three base seam between parts, which in the case of fusion proteins, encodes a relatively innocuous alanine residue. The inner end of each half-Clip bears an extension, which exists only to prevent the blunt ends from ligating (Supplementary Figure S1).

PaperClip libraries are prepared by obtaining four oligonucleotides for each part, phosphorylation using T4 Polynucleotide Kinase, and annealing to form two pairs (half-Clips), ‘Upstream’ and ‘Downstream’ (Figure 1A). If it is desired that a particular DNA part should be usable in either orientation, two additional versions of oligonucleotides UF and DR should also be obtained, exchanging the external overhangs. Clip oligonucleotides can then be stored with the part and used in any future assembly involving that part. Essentially, for each assembly, a set of ligations is performed, in which the upstream half-Clip for each part is ligated to the Downstream half-Clip for the adjacent part to form the final Clip. These ligations are highly efficient with one hour incubation, and do not require purification (Figure 1B).

### Insertion of a short intervening sequence between two parts

Short sequences of intervening DNA, such as ribosome binding sites, or sequences encoding fusion protein linkers or N- or C-terminal tags, can be added between two half-Clips (Figure 1C and D). The DNA of interest should be obtained as four oligonucleotides. As for Clips, the additional sequence should contain a GCC overhang on the upstream forward oligonucleotide and GGC overhang on the downstream reverse oligonucleotide. After phosphorylation, the annealed pairs of oligonucleotides should create 5 base complementary sticky ends (to restore the sequence of interest). Ligation to generate the Clip then occurs in two steps. The Downstream half is ligated with the upstream half of the intervening sequence in one tube, and the downstream half of the intervening sequence is ligated with the Upstream half-Clip of the following part in a second tube. Ligation is performed as described in ‘Materials and Methods’ section. After 1 h incubation at 16°C the content of the two tubes are mixed together and incubated for further 1 h at 16°C. The efficiency of ligation is suitable for the next assembly step. In this study we have tested intervening DNA of 60 and 100 bp.

### Overview of assembly

To summarize, a library of parts suitable for PaperClip assembly consists of the parts themselves plus four oligonucleotides of ∼40 bases for each part. These are phosphorylated and annealed to form two half-Clips for each part, which are stored with the part. Prior to assembly, adjacent half-Clips are ligated to form Clips, which can be stored and re-used for future assemblies. The Clips thus formed are then mixed with the parts and used for assembly using any of a number of standard methods (Figure 1E). The order of the parts in the final construct can be changed by using different Clips, with pre-designed order of the half-Clips (Figure 1E). Only 24 oligonucleotides were used in this study for assembly of plasmids containing up to six DNA parts (Supplementary Table S1). Twelve of these oligonucleotides (UF and DR) were used to amplify six DNA parts used in this study (Supplementary Figure S2).

### Assembly using PCR

**Figure 2.**
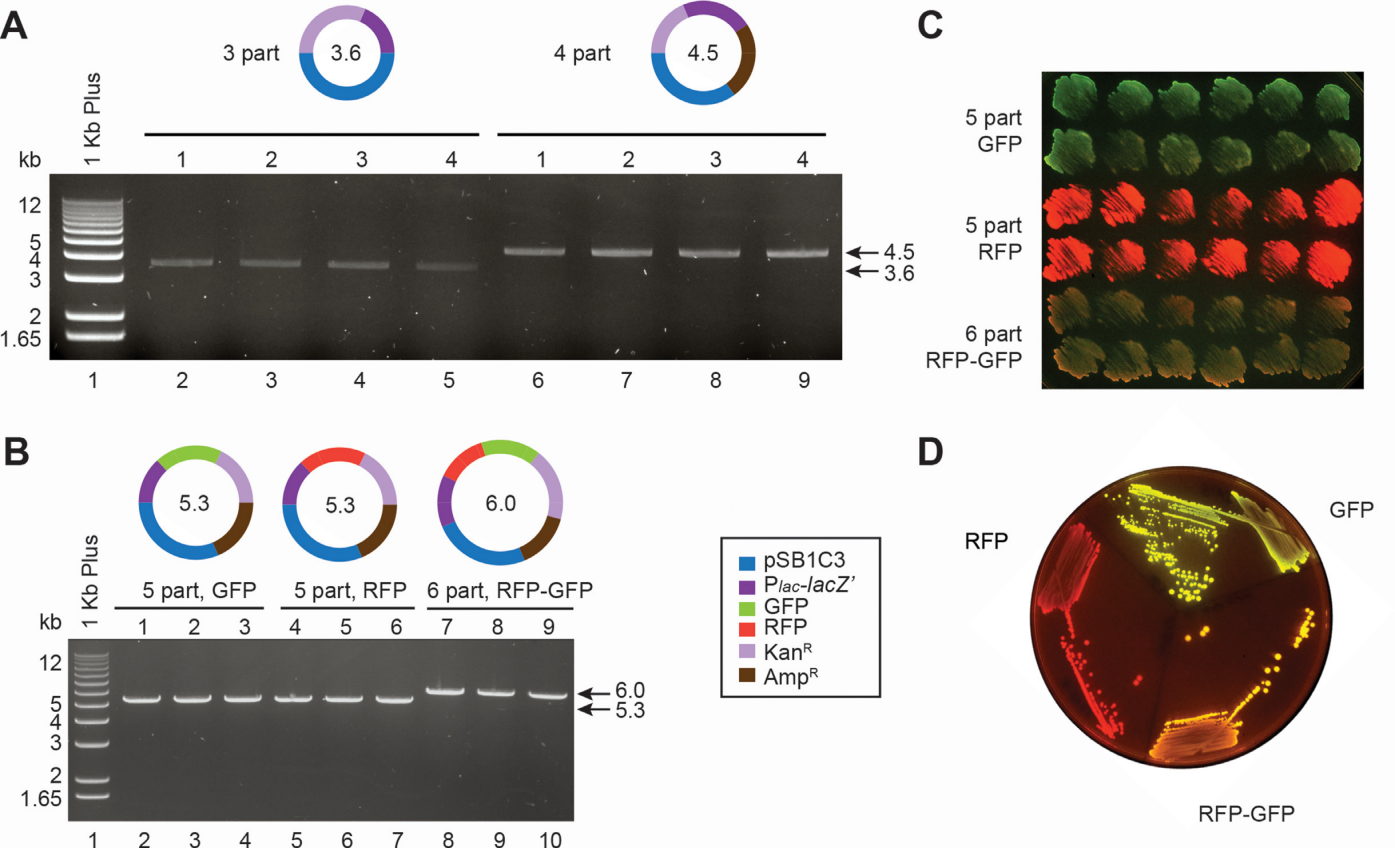
Analysis of the products from three-, four-, five- and six-part assemblies. (**A**) Restriction digest of the plasmid DNA from four kanamycin resistant colonies from three-part (3746 colonies in total) and four-part (1206 colonies in total) assemblies. All clones contained the plasmid of the expected sizes—3.6 kb for three-part and 4.5 kb for four-part assembly. (**B**) Schemes of the plasmids assembled from five and six DNA parts (pSB1C3 backbone, P*_lac_*-*lacZ*′, GFP, RFP, kanamycin resistance cassette—Kan^R^ and ampicillin resistance cassette—Amp^R^) and restriction digest of the plasmid DNA isolated from three randomly selected kanamycin resistant clones. The expected product size is 5.3 kb for five-part and 6.0 kb for six-part assembly. (**C**) Twelve randomly picked kanamycin resistant clones were patched on LB agar contacting IPTG to induce the expression of GFP and/or RFP. (**D**) One clone from each assembly was streaked out to single colonies to confirm purity of the culture.

The best results for the PaperClip assembly we have obtained using a modified version of the CPEC method (20), two-step PCR. During the first stages of PCR the DNA parts are amplified with Clips acting as primers, generating homology to the neighbouring parts. Later cycles of the PCR will be equivalent to CPEC assembly, resulting in a circular product containing nicks, which are repaired by the host cells. The main advantage of PaperClip assembly is that acquisition of end-homology and assembly are performed during one reaction. We have successfully assembled up to six parts using two-step PCR. From three to four colonies were analyzed by restriction digests of the plasmid DNA (Figure 2A) as well as by sequencing. Representative results of five- and six-part assemblies are shown in Figure 2B–D. Twelve randomly chosen colonies were patched on agar plates containing IPTG to induce expression of GFP and/or RFP and streaked out to confirm the purity of the culture (Figure 2C and D). The functionality of all assembled genes was confirmed by growth on agar containing appropriate antibiotics. All tested constructs were assembled correctly as confirmed by Sanger sequencing.

We have observed accumulation of the desired product size visualized on agarose gels by the 20th cycle of the PCR reaction (Supplementary Figure S3A) and optimized the volume of the PCR reaction to be transformed for maximizing the number of resistant colonies (Supplementary Figure S3B).

**Figure 3.**
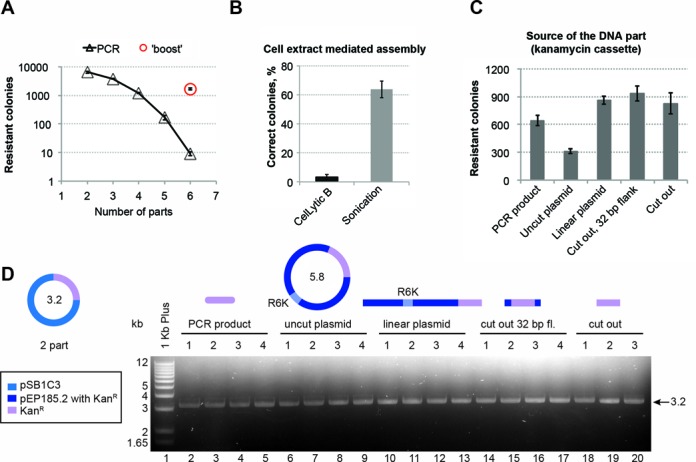
Optimization of PaperClip efficiency. (**A**) The efficiency of assembly decreases with the increase of the number of parts. For six-part assemblies, a second ‘boost’ PCR reaction gave a 150-fold increase in the number of kanamycin-resistant colonies. (**B**) Two methods of cell extract preparation were compared in PaperClip assembly. In this two-part assembly P*_lac_*-*lacZ*′ cassette was cloned into pSB1C3 backbone. The correct colonies are blue (when plated on LB agar containing IPTG and X-Gal), while the colonies containing initial pSB1C3 plasmid will be red (containing RFP cassette). Extracts after sonication of the *Escherichia coli* DH10B Red/ET cells showed 20× higher efficiency in the assembly as judged by the percentage of blue colonies. (**C**) Assembly of the pSB1C3 backbone and kanamycin resistance (Kan^R^) cassette, where the resistance cassette was represented in five forms: as a PCR product (641 colonies), uncut plasmid (contains R6K conditional origin of replication and can not replicate in the destination strain, 311 colonies), linearized plasmid (863 colonies), cut out with 32 bp flanking regions (935 colonies) and cut out precisely (827 colonies). Number of colonies is averaged from three plates when 1 μl of the assembly was transformed and 100 μl (of 500 μl) plated out. (**D**) Plasmid DNA from four and three random kanamycin resistant clones was isolated and subjected to restriction digest. All clones contained plasmid DNA of the expected size of 3.2 kb.

The number of kanamycin resistant colonies observed after assembly decreases with increasing number of parts in the assembly (Figure 3A). Increasing the number of cycles during PCR did not result in increased numbers of resistant colonies.

### ‘Boost’ PCR

We hypothesized that during assembly of large constructs (>5 kb), extra dNTPs may be required. To test this, 5 μl of the first assembly PCR reaction were used as template for a subsequent two-step primer-less PCR reaction (see ‘Materials and Methods’ section for further details), which resulted in a ‘boost’ of the number of resistant colonies from 10 to >1000 (Figure 3A). Less than 1% of the observed colonies lacked either GFP or RFP as judged by phenotype. Twelve randomly chosen colonies for each of the five- and six-part assemblies had the correct phenotype (Figure 2C) and three sequenced constructs from each assembly contained no mutations.

### Assembly using cell extracts

The PaperClip procedure can also be used with other assembly methods. As an example, we have also performed two and three part assemblies using cell extracts from *E. coli* DH10B cells expressing the Lambda Red/ET system (GeneBridges). We have compared two methods of cell extract preparation—sonication and chemical lysis (CelLyticB, Sigma) (see ‘Materials and Methods’ section for more details). In this experiment we assembled a P*_lac_*-*lacZ*′ cassette and pSB1C3 backbone (conferring chloramphenicol resistance). The plasmid donor of the pSB1C3 backbone contains an RFP cassette, so that the background colonies will be red, while the correctly assembled colonies will turn blue when plated on agar containing IPTG and X-gal (21). Assembly with the cell extract prepared by sonication (19) results in 20× more blue (correctly assembled) colonies, than with the cell extract prepared by chemical lysis (Figure 3B). This may be due to the composition of the buffer in which cells are resuspended prior to lysis (or during lysis for CelLyticB) or the efficiency of cell wall disruption. The resulting constructs were subjected to restriction analysis (Supplementary Figure S4A and B) and sequenced to confirm the correct order of the DNA parts as well as presence of the characteristic GCC seams. Using cell extracts we have successfully assembled two constructs containing three DNA parts in different order (Supplementary Figure S3C and D). Sequencing confirmed the correct order of the DNA parts, absence of mutations and presence of GCC seams.

In principle, similar Clips should also be able to direct assembly by methods such as Gibson assembly (2,16) and ligase chain reaction (LCR) (15,22). If Clip oligonucleotides are used to amplify the DNA parts, the UF primer introduces ‘GCC’ at the upstream end and the DR primer adds ‘GGC’ at the downstream end of the PCR product. Therefore, if LCR assembly is to be used, initial part amplification must be performed using a version of either primer UF or primer DR which does not include these three bases, in order to avoid a three-base overlap which would prevent LCR assembly.

### Form of the DNA parts

In the assemblies described above we have used DNA parts in the form of PCR products (Supplementary Figure S1). We have tested efficiency of assembly of DNA in different forms—PCR product, linearized plasmid, undigested plasmid, the part excised from a plasmid with restriction enzymes precisely at the ends of the part, or excised with 32 bp flanking DNA (Supplementary Figure S2C). For this two-part PCR assembly, the test gene was a kanamycin resistance cassette, which was initially present in a plasmid vector with conditional origin of replication R6K. This origin prevents the kanamycin resistant plasmid from replication in the recipient strain *E.coli* DH10B. Results shown in Figure 3C indicate that all of the tested forms of DNA are suitable for PaperClip assembly, with the lowest efficiency observed for uncut plasmid DNA, and the highest with the gene excised from the plasmid. From three to four randomly picked kanamycin colonies were used for plasmid DNA isolation and subjected to restriction digest. All of the analyzed plasmid DNA had correct size (Figure 3D) and accuracy of assemblies was confirmed by Sanger sequencing (http://archive.bio.ed.ac.uk/papers/paperclip/). Notably, no DNA purification step was performed prior to assembly (see ‘Materials and Methods’ for more details), which should allow straightforward automation.

## DISCUSSION

The key advantage of PaperClip over other assembly methods is that existing part libraries can immediately be used for multi-part single pot assembly without re-cloning or amplification. The only requirement is to obtain four short oligonucleotides per part, which can then be stored and used in any assembly involving that part. Internal restriction sites do not pose any problem. PaperClip does not require expensive enzymes or kits; three enzymes, polynucleotide kinase, T4 DNA ligase and a proofreading polymerase, are the only enzymes needed for assembly, thus reducing the cost and time of the assemblies.

Since PaperClip assembly is homology based, the final constructs cannot contain repetitive parts or more than 40 bases of identical regions, as during amplification or recombination, the regions between the duplications will be spliced out. It should also be noted that final constructs after PaperClip assembly will contain a GCC tri-nucleotide seam (‘scar’) between each part. Thus PaperClip is not a ‘scarless’ assembly method, and may not be suitable for some applications where complete control of all bases is required. From a practical point of view, three bases is the minimum number of nucleotides, which can be used to prevent frame shift in protein fusions. GCC encodes alanine, which should be relatively innocuous in generation of fusion proteins. If necessary for particular applications, the GCC seam can be changed to any user-defined nucleotides to meet the requirements of the design. For example, a modified form of PaperClip, ‘BrickClip’ (BBF RFC104), using CTA seams, can be used for multi-part BioBrick assembly, generating the same products that would be created by standard RFC10 assembly.

The advantage of PaperClip over other methods utilizing bridging oligonucleotides is that there is no need to obtain new oligonucleotides for each assembly involving a different order of parts. This reduces cost and preparation time for assemblies. If re-using pre-made Clips, the assembly reaction takes ∼1–2 h depending on the final construct size. A number of different assemblies can be performed in parallel, as the two-step PCR programme for the assembly is universal. If re-circularization of a single part is required, simple ligation of the Downstream half-Clip of one part with the Upstream half-Clip of the same part might be performed, followed by assembly (either by PCR or using cell extracts).

PaperClip assembly is also easily amenable to automation, since gel purification steps are not required. By allowing rapid, simple one-pot assembly of multiple parts from existing collections present in any laboratory without requirement for expensive enzymes, PaperClip assembly will facilitate the adoption of synthetic biology techniques by the wider bioscience community.

## SUPPLEMENTARY DATA

Supplementary Data are available at NAR Online.

SUPPLEMENTARY DATA
