# Supplementary material for: PaperClip: rapid multi-part DNA assembly from existing libraries
Source: Nucleic Acids Res. 2014 Sep 8;42(20):e154. doi: 10.1093/nar/gku829 (PMC4227759; doi:10.1093/nar/gku829)
Supplement: SUPPLEMENTARY DATA [file supp_42_20_e154__index.html]

PaperClip: rapid multi-part DNA assembly from existing libraries — PaperClip: rapid multi-part DNA assembly from existing libraries — SUPPLEMENTARY DATA 

# PaperClip: rapid multi-part DNA assembly from existing libraries

## SUPPLEMENTARY DATA

**Files in this Data Supplement:**

- SUPPLEMENTARY DATA
