# Supplementary material for: PaperClip: rapid multi-part DNA assembly from existing libraries
Source: Nucleic Acids Res. 2014 Sep 8;42(20):e154. doi: 10.1093/nar/gku829 (PMC4227759; doi:10.1093/nar/gku829)
Supplement: SUPPLEMENTARY DATA [file supp_gku829_nar-02044-met-h-2014-File005.pdf]

Supplementary information

PaperClip: rapid multi-part DNA assembly from existing libraries

Maryia Trubitsyna, Gracjan Michlewski, Yizhi Cai, Alistair Elfick and Christopher E. French

Supplementary Table 1. List of 24 oligonucleotides used to perform all the assemblies in this study. Upstream Forward and Downstream Reverse oligonucleotides were also used for amplification of the corresponding parts from the template DNA.

pSB1C3 backbone (from BBa_K1122005)	Upstream Forward	GCCtccggcaaaaaagggcaaggtgtcaccaccctgcccttttt
	Upstream Reverse	agggcagggtggtgacaccttgcccttttttgccgga
	Downstream Forward	ggcagaatttcagataaaaaaatccttagctttcgc
	Downstream Reverse	GGCgcgaaagctaaggatttttttatctgaaattctgcctcg
pLac-LacZ (synthetic)	Upstream Forward	GCCtctagattcggagtgagcgcaacgcaattaatgtgagttagctcac
	Upstream Reverse	agctaactcacattaattgcgttgcgctcactccgaatctaga
	Downstream Forward	ccagaagcggtggccggaaagctggctggatgagcctggatcc
	Downstream Reverse	GGCggatccaggctcatccagccagctttccggccaccgcttctggtgc
Kanamycin resistance cassette (from pUC4K, cloned into pEP185.2)	Upstream Forward	GCCcgctgaggtctgcctcgtgaagaaggtgttgctgactcat
	Upstream Reverse	gtcagcaacaccttcttcacgaggcagacctcagcg
	Downstream Forward	aacatcagagattttgagacacaacgtggctttccc
	Downstream Reverse	GGCgggaaagccacgttgtgtctcaaaatctctgatgttacat
Ampicillin resistance cassette (from pBSKS(+))	Upstream Forward	GCCttaccaatgcttaatcagtgaggcacctatctcagcgatc
	Upstream Reverse	cgctgagataggtgcctcactgattaagcattggtaa
	Downstream Forward	cagggttattgtctcatgagcggatacatatttgaa
	Downstream Reverse	GGCttcaaatatgtatccgctcatgagacaataaccctgataa
GFP (from BBa_I13522)	Upstream Forward	GCCaggaaacagctatgtcgcgtaaaggagaagaacttttcac
	Upstream Reverse	aaagttcttctcctttacgcgacatagctgtttcct
	Downstream Forward	attacacatggcatggatgaactatacaaagcctaa
	Downstream Reverse	GGCttaggctttgtatagttcatccatgccatgtgtaatccca
RFP (from BBa_K1122005)	Upstream Forward	GCCaggaaacagctatggcttcctccgaagacgttatcaaag
	Upstream Reverse	gataacgtcttcggaggaagccatagctgtttcct
	Downstream Forward	cgaacgtgctgaaggtcgtcactccaccggtgcttaa
	Downstream Reverse	GGCttaagcaccggtggagtgacgaccttcagcacgttcgtac

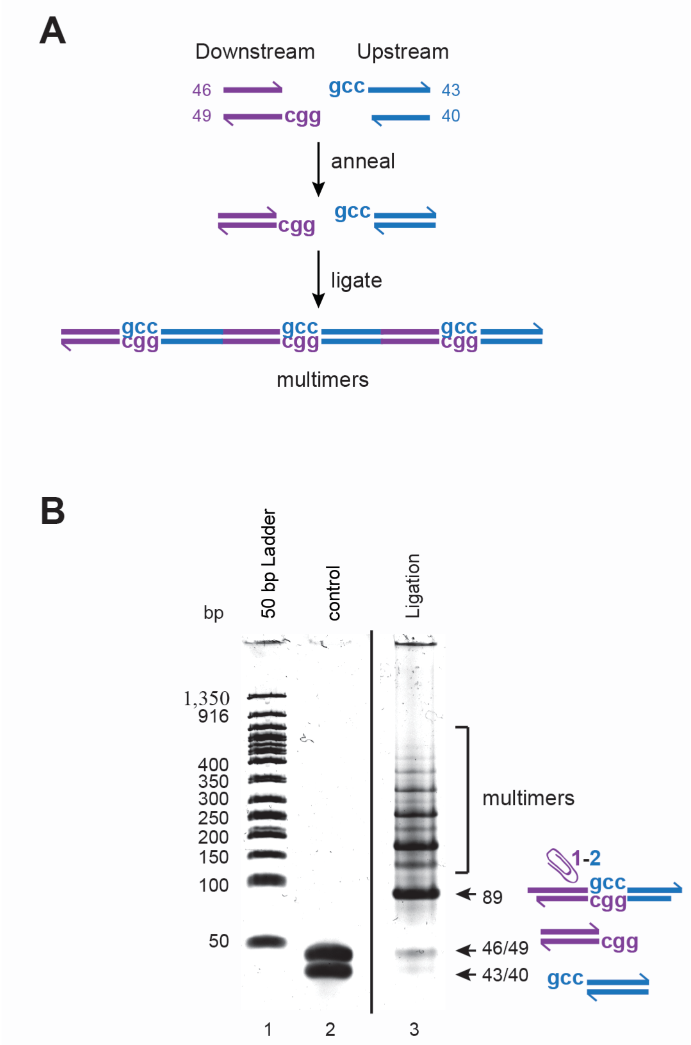

Supplementary Figure 1: Scheme of preparation of the Clips bearing blunt ends in the end facing the DNA part (A). Undesired over-ligation of blunt ends is efficient enough to create multimers even after one hour incubation (B)

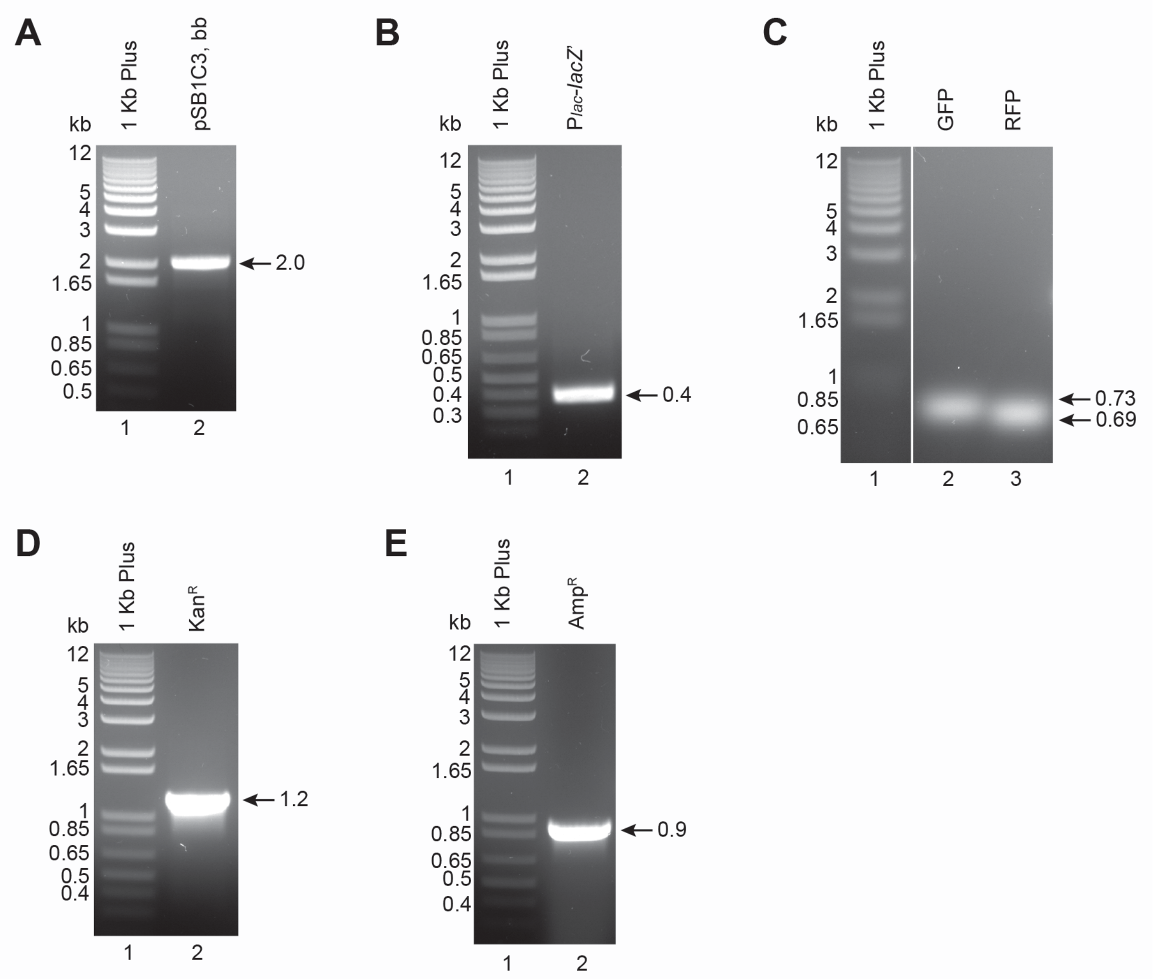

Supplementary Figure 2: Six genes used in this studies have been amplified from plasmid DNA template using Upstream Forward and Downstream Reverse oligonucleotides as primers. (A) pSB1C3 backbone of 2.0 kb was amplified from BBa_K1122005 (Registry of Standard Biological Parts). (B) P_{lac}-*lacZ'* cassette was amplified from a plasmid containing artificially synthesized cassette (laboratory collection). (C) GFP and RFP parts were amplified from BBa_I13522 and BBa_K1122005 respectively. (D) Kanamycin resistance cassette was amplified from pEP185.2 plasmid with Kan^R cloned in it (laboratory collection). (E) Ampicillin cassette was amplified from pBSKS(+) cloning vector.

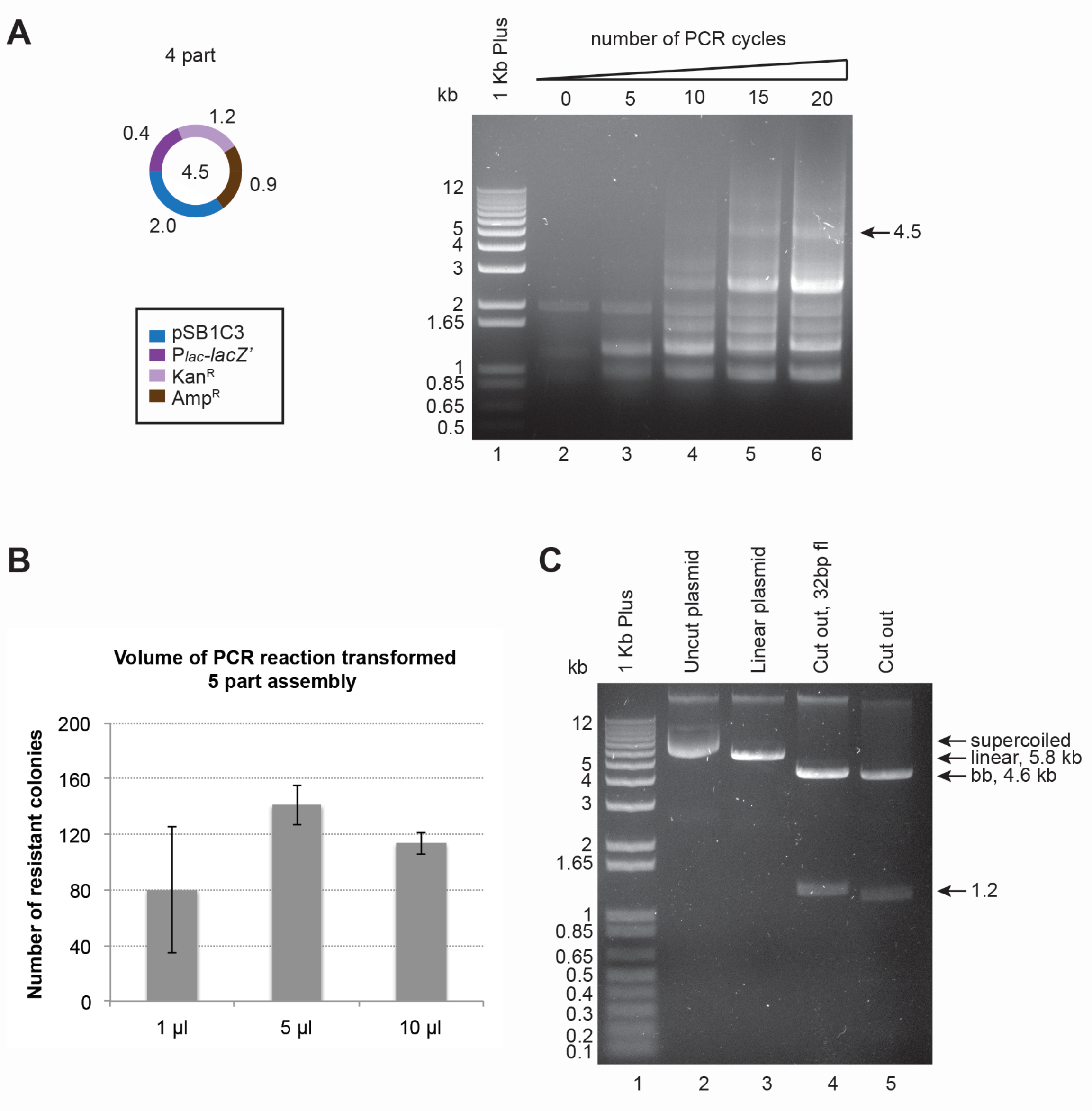

Supplementary Figure 3: Optimisation of the PaperClip assembly conditions by PCR. (A) The expected product of four part assembly (4.5 kb) becomes visible on agarose gel after the 20th cycle. (B) Optimisation of the transformation volume. The maximum number of colonies for 5 part assembly was observed when 5 μl of the PCR reaction (1/10th) was transformed into 100 μl of chemically competent cells *E.coli* DH10B. The 2/5th of the recovery volume (40%) was plated out on agar plates containing selective antibiotic. (C) Preparation of different forms of DNA part (kanamycin resistance cassette) by restriction digest.

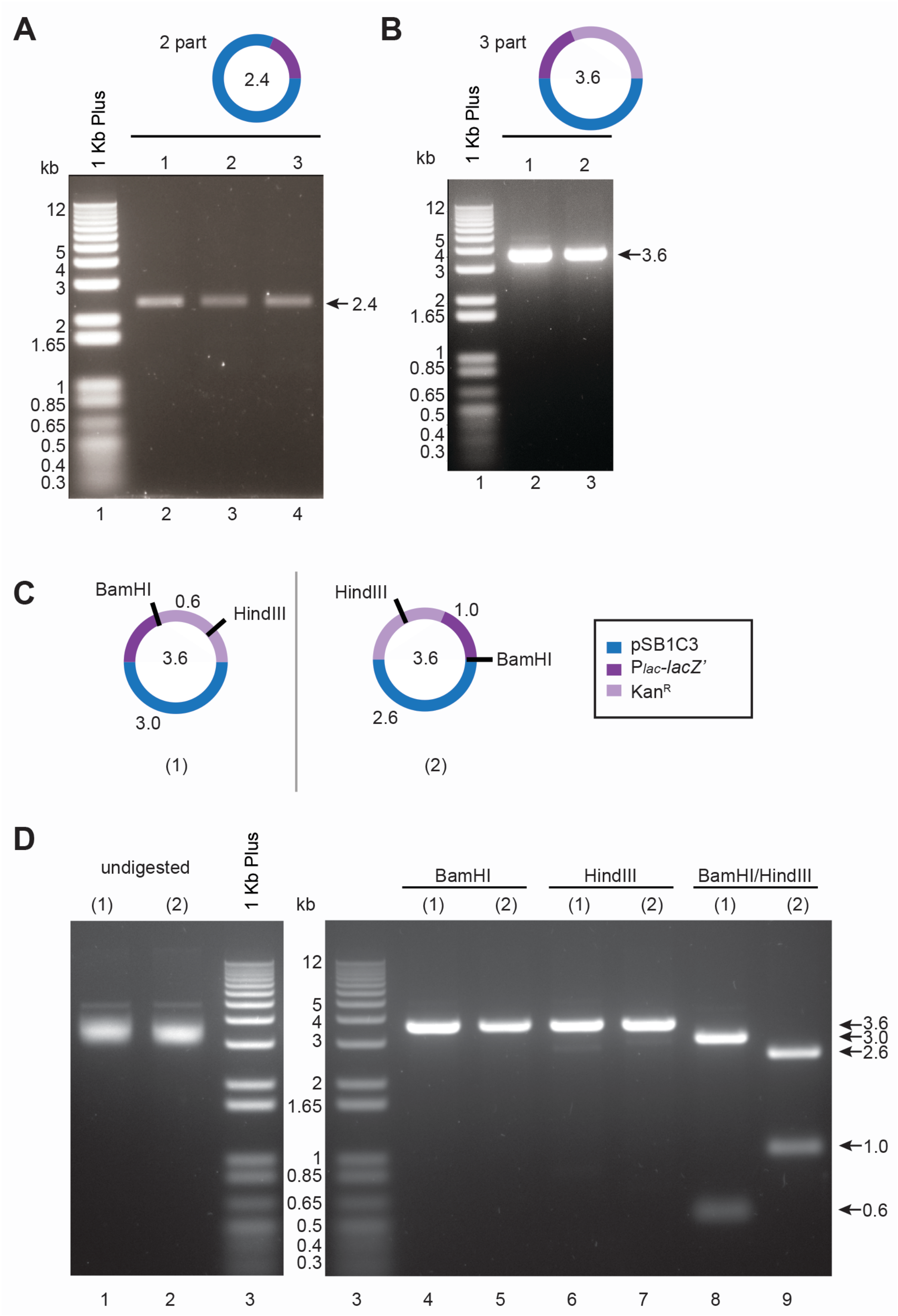

Supplementary Figure 4: Assembly using cell extracts. Assembly of two parts (A) and three parts (B) facilitated by the recombination enzymes present in *E.coli* DH10B Red/ET cell extract. Restriction digest of the isolated plasmids represents the expected sizes of the final DNA constructs – 2.4 kb for two part and 3.6 kb for three part assembly. (C) Scheme of the three part

assembly with alternative parts order. pSB1C3, P_{lac}-*lacZ’* and kanamycin resistance cassette were assembled in two forms. (D) Analytical restriction digest of plasmid DNA from two blue colonies. The restriction digest pattern of double digest is correct (lanes 8 and 9). These plasmids were sequenced to confirm the presence of characteristic GCC seams and absence of mutations.
